# Intensive care discharge delay is associated with increased hospital length of stay: A multicentre prospective observational study

**DOI:** 10.1371/journal.pone.0181827

**Published:** 2017-07-27

**Authors:** Ravindranath Tiruvoipati, John Botha, Jason Fletcher, Himangsu Gangopadhyay, Mainak Majumdar, Sanjiv Vij, Eldho Paul, David Pilcher, the Australia and New Zealand Intensive Care Society (ANZICS) Clinical Trials Group

**Affiliations:** 1 Department of Intensive Care Medicine, Frankston Hospital, Frankston, Victoria, Australia; 2 School of Public Health, Faculty of Medicine, Nursing and Health Sciences, Monash University, Melbourne, Victoria, Australia; 3 Bendigo Hospital, Bendigo, Victoria, Australia; 4 Box Hill Hospital, Box Hill, Victoria, Australia; 5 Maroondah Hospital, Ringwood East, Victoria, Australia; 6 Dandenong Hospital, Dandenong, Victoria, Australia; 7 Department of Epidemiology and Preventive Medicine, School of Public Health and Preventive Medicine, Monash University, Melbourne, Victoria, Australia; 8 Clinical Haematology Department, The Alfred Hospital, Melbourne, Victoria, Australia; 9 Department of Intensive Care Medicine, Alfred Hospital, Melbourne, Victoria, Australia; Azienda Ospedaliero Universitaria Careggi, ITALY

## Abstract

**Background:**

Some patients experience a delayed discharge from the intensive care unit (ICU) where the intended and actual discharge times do not coincide. The clinical implications of this remain unclear.

**Objective:**

To determine the incidence and duration of delayed ICU discharge, identify the reasons for delay and evaluate the clinical consequences.

**Methods:**

Prospective multi-centre observational study involving five ICUs over a 3-month period. Delay in discharge was defined as >6 hours from the planned discharge time. The primary outcome measure was hospital length stay after ICU discharge decision. Secondary outcome measures included ICU discharge after-hours, incidence of delirium, survival to hospital discharge, discharge destination, the incidence of ICU acquired infections, revocation of ICU discharge decision, unplanned readmissions to ICU within 72 hours, review of patients admitting team after ICU discharge decision.

**Results:**

A total of 955 out of 1118 patients discharged were included in analysis. 49.9% of the patients discharge was delayed. The most common reason (74%) for delay in discharge was non-availability of ward bed. The median duration of the delay was 24 hours. On univariable analysis, the duration of hospital stay from the time of ICU discharge decision was significantly higher in patients who had ICU discharge delay (Median days-5 vs 6; p = 0.003). After-hours discharge was higher in patients whose discharge was delayed (34% Vs 10%; p<0.001). There was no statistically significant difference in the other secondary outcomes analysed. Multivariable analysis adjusting for known confounders revealed delayed ICU discharge was independently associated with increased hospital length of stay.

**Conclusion:**

Half of all ICU patients experienced a delay in ICU discharge. Delayed discharge was associated with increased hospital length of stay.

## Introduction

Discharge from intensive care unit (ICU) is usually planned when patients have recovered from critical illness or when the intensive care physician believes that further intensive care treatment is no longer in the patients best interest. The duration of stay in intensive care depends on the severity of the presenting problem and the pre-morbid physiological reserve of the patient. It is known from the published literature that admission and discharge of patients from intensive care units during ‘after-hour’ periods is associated with an increased mortality and morbidity[1–3].

Timely discharge of patients from the ICU is important. The non-availability of beds in the hospital wards may contribute to ICU discharge delay. It is essential that patients are discharged in a timely manner to facilitate their ongoing care and hospital discharge planning. The Australian Council on Health Care Standards (ACHS) recommended a delay in discharge of more than 6 hours from ICU to be a key performance indicator for acute hospital service delivery [4].

Delayed discharge from ICU may have undesirable patient consequences. The extended stay of patients in intensive care units may be associated with potential problems including disturbances in sleep[5], increased incidence of delirium due to poor quality of sleep in ICU[6], increased risk of acquiring multi-resistant bacterial infections[7], and a delayed review or irregular reviews from the parent (admitting) team of the patient, after the discharge from ICU and an overall increase in duration of hospital stay. Furthermore delay in discharge may also delay or reduce the availability of ICU facilities for those patients in need of intensive care.

Our aim was to measure the incidence and reasons for delayed ICU discharge and assess its impact on the patient outcomes. Our hypothesis was that a delay in ICU discharge may be associated with an increased hospital length of stay from the time of ICU discharge decision.

## Methods

Approval from the Human Research Ethics Committees (HREC) of each participating hospitals was obtained prior to commencement of this study. Consent from individual patients was waived. Identified patient level data was available to authors at their own individual site but was collated into a single de-identified dataset for analysis and reporting purposes.

### Study Design

**Fig 1:**
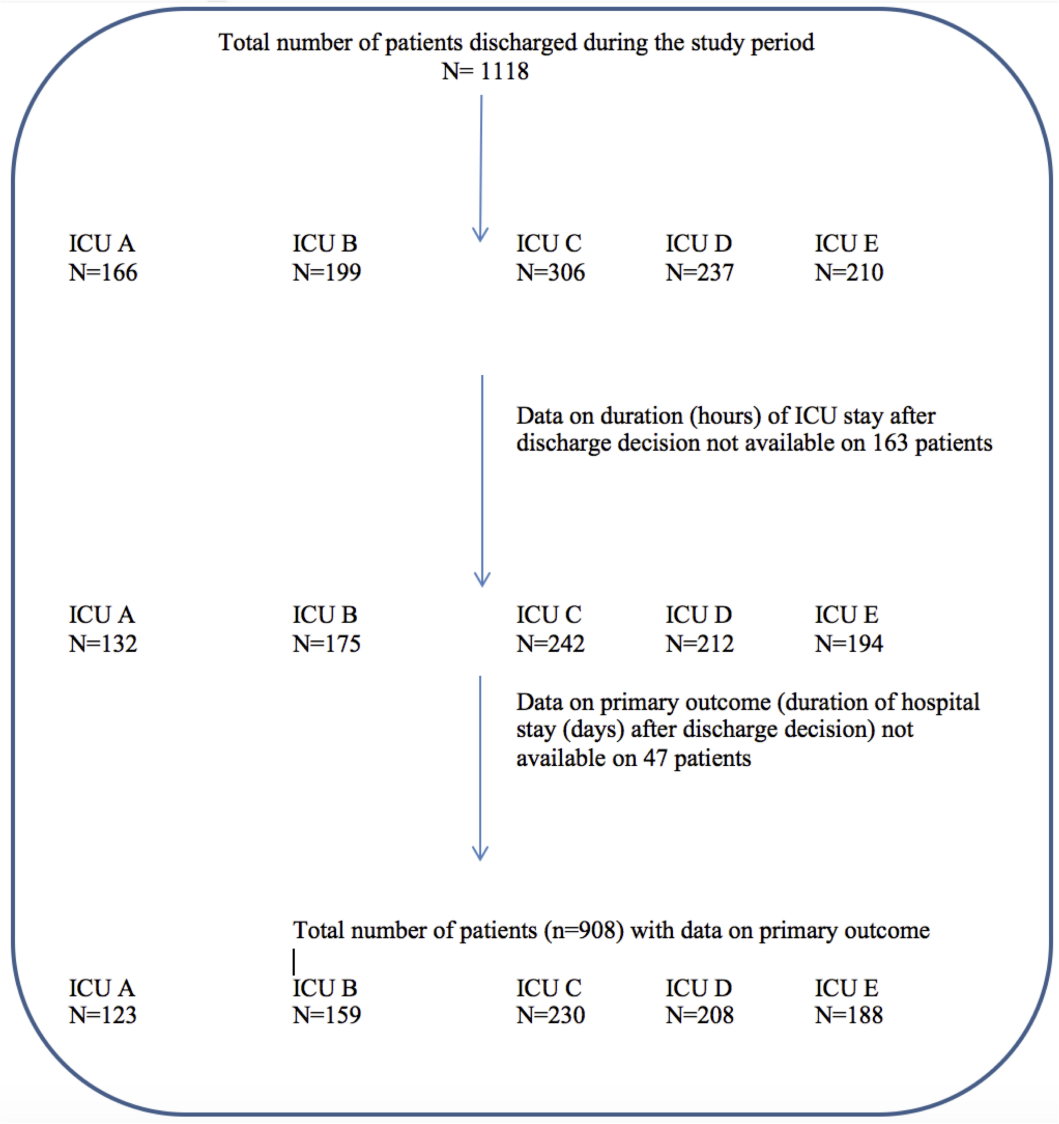
Description of patient recruitment.

This study was a prospective observational study performed over a 3-month period (Feb 2013-May 2013) involving the ICUs of five hospitals (Bendigo Hospital, Box Hill hospital, Dandenong Hospital, Frankston Hospital and Maroondah Hospital) in Victoria, Australia. No ICU in this study admitted cardiac and neurosurgical patients or major trauma patients. Patients who deemed suitable to be discharged from the ICU were enrolled into the study. Patients were considered suitable to be discharged from ICU if they no longer required organ support or advanced monitoring such as arterial, central venous or pulmonary arterial pressures or cardiac output. Suitability of discharge was determined by the intensive care specialist and this remained consistent across the participating intensive care units. Discharge decisions were predominantly made during the first ICU ward rounds performed by the intensivist. The exact times of ICU discharge decision and the actual time of ICU discharge were documented on the patients’ charts. If the time of discharge decision or the time of discharge was not documented or were unclear patients were excluded (Fig 1). The intensive care specialists were not aware of the ward bed availability for patients to be discharged at the time of making discharge decisions. After enrolment patients were categorised into two groups based on their time taken to be discharged from the ICU.

### No delay in discharge group (No delay)

Discharged within 6 hours of the decision to discharge from ICU and.

### Delayed discharge group (Delayed)

Discharged at least 6 hours after the decision to discharge from ICU was made.

### Inclusion Criteria

All patients assessed as suitable to be discharged from the ICU by the intensivist in charge of the intensive care unit.

### Exclusion Criteria

There were no general exclusion criteria for patients. However, the following patients were excluded from assessment of delirium using the Confusion Assessment Method for the ICU (CAM ICU): Patients with inability to communicate due to severe dementia or severe hearing loss or brain injury or patients who were discharged for comfort / palliative care.

### Outcome measures

The primary outcome measure included the Duration of hospital stay from the time of ICU discharge decision. Secondary outcome measures included discharge from ICU after hours (18:00 to 08:00), Incidence of delirium from ICU discharge decision time to the first 48 hours of admission to a hospital ward, survival to hospital discharge, discharge destination (including home, rehabilitation, other hospital or death), the incidence of infections acquired in intensive care after the decision to discharge from ICU was made, revocation of ICU discharge decision, unplanned readmissions to ICU within 72 hours, review of patients by parent (admitting) team after ICU discharge decision.

The incidence of delirium was assessed by the Confusion Assessment Method for the ICU (CAM-ICU)[8, 9] by an independent person who was trained to assess delirium but was not an investigator of this study. Delirium was assessed at least once daily starting from the time of planned ICU discharge. First assessment of delirium was done within 6 hours of planned ICU discharge decision time. Daily delirium assessments were performed until 48 hours in the hospital ward. Any episodes of delirium that occurred in between the daily assessment from CAM ICU were also recorded and included in the analysis.

For the purpose of this study ICU acquired infection was defined as new infection occurring within 48 hours of the ICU discharge. Sepsis was defined according to 2001 SCCM/ESICM/ACCP/ATS/SIS International Sepsis Definitions Conference in identifying patients with sepsis[10]. Data was collected by a designated data collector on a specifically designed data collection sheet.

### Statistical analysis

Categorical variables were presented using frequencies and percentages. Continuous variables were summarised using means and standard deviations or medians and interquartile ranges depending on the underlying distribution of the data. Univariable comparisons between groups (delayed discharge: yes vs no) were made using Student’s t-test for normally distributed continuous variables, Mann-Whitney U test for non-normally distributed continuous variables and chi-square test for equal proportions or Fisher’s exact test as appropriate for categorical variables. The distribution of the primary outcome duration of hospital stay from the time of ICU discharge decision was found to be approximately normal after logarithmic transformation. Multivariable analysis was performed using linear regression to assess the independent effect of delayed ICU discharge with results reported as geometric means and 95% confidence intervals (95% CI). Variables with p<0.05 on univariable analysis or those judged to be clinically significant were included as covariates in the regression analysis. The variables that were included in multivariable analysis were APACHE III score (with age component removed), age, duration of ICU stay after the ICU discharge decision was made, requirement of mechanical ventilation, admitting unit, centre where the patients was admitted, and isolation room requirement at ICU discharge. A two-sided p value less than 0.05 indicated statistical significance. Analyses were performed with SAS software version 9.4 (SAS Institute, Cary, NC, USA).

## Results

A total of 1118 patients were discharged from the participating ICUs during the study period. Of these, 955 patients (85%) were included in the final analysis. A description of patient recruitment is presented in Fig 1. Data on primary outcome was available in 908 patients (95%). The demographic and outcome characteristics are presented in Table 1. Overall, patients who had a delay in ICU discharge were older and had a higher SAPS II and APACHE III scores on admission (Table 1).

**Table 1:** Comparison of patient characteristics between patients with and without delay in discharge at the time of admission to intensive care units.

Variable	Discharged with no delay (N = 478)	Discharged with delay(N = 477)	P Value
**Age (mean, SD)**	59.7 (20) (n = 472)	63.2 (17.7) (n = 469)	0.005
**Age range**	12–96	17–93	
**Sex Male**	52% (n = 246/473)	50% (n = 234/467)	0.52
**Surgical Admission**	47% (n = 224/476)	46% (n = 217/472)	0.74
**Emergency Admission**	75% (n = 350/467)	79% (n = 372/471)	0.21
**Ventilated during ICU Admission (NIV &IV)**	32% (n = 150/470)	38% (n = 177/466)	0.08
**Invasively ventilated**	68% (n = 80/118)	69% (n = 98/142)	0.95
**COPD**	19% (n = 70/367)	16% (n = 65/404)	0.37
**Diabetes**	22% (n = 103/466)	24% (n = 114/473)	0.47
**Hypertension**	40% (n = 187/467)	43% (n = 203/472)	0.36
**Chronic renal failure**	10% (n = 46/464)	8% (n = 38/470)	0.28
**Ischaemic heart disease**	23% (n = 107/465)	22% (n = 104/471)	0.73
Other Comorbidities*	n = 50	n = 40	
**SAPS II (median, IQR)**	26 (18–37) (n = 361)	30.5 (21–42)(n = 390)	< 0.001
**APACHE III score (mean, SD)**	49.5 (22.7) (n = 361)	54.6 (24.4) (n = 390)	0.003
**APACHE III score (mean, SD) without age component (Mean, SD)**	39.5 (20.9) (n = 361)	43.7 (23.6) (n = 390)	0.01

COPD: Chronic obstructive pulmonary disease; SAPS: *Simplified Acute Physiology Score; APACHE*: *Acute Physiology and Chronic Health Evaluation*

*Other comorbidities included, Atrial fibrillation, congestive cardiac failure, hypercholesterolemia, Aortic stenosis, Asthma, bronchiectasis, pulmonary hypertension, pulmonary fibrosis, obstructive sleep apnoea, morbid obesity, inflammatory bowel disease, chronic liver failure, immunosuppression, metastatic carcinoma, acquired brain injury, stroke, quadriplegia, Alzheimer’s disease, multiple sclerosis, depression, schizophrenia, hypothyroidism, psoriasis, gout, systemic lupus erythematosus

**Fig 2:**
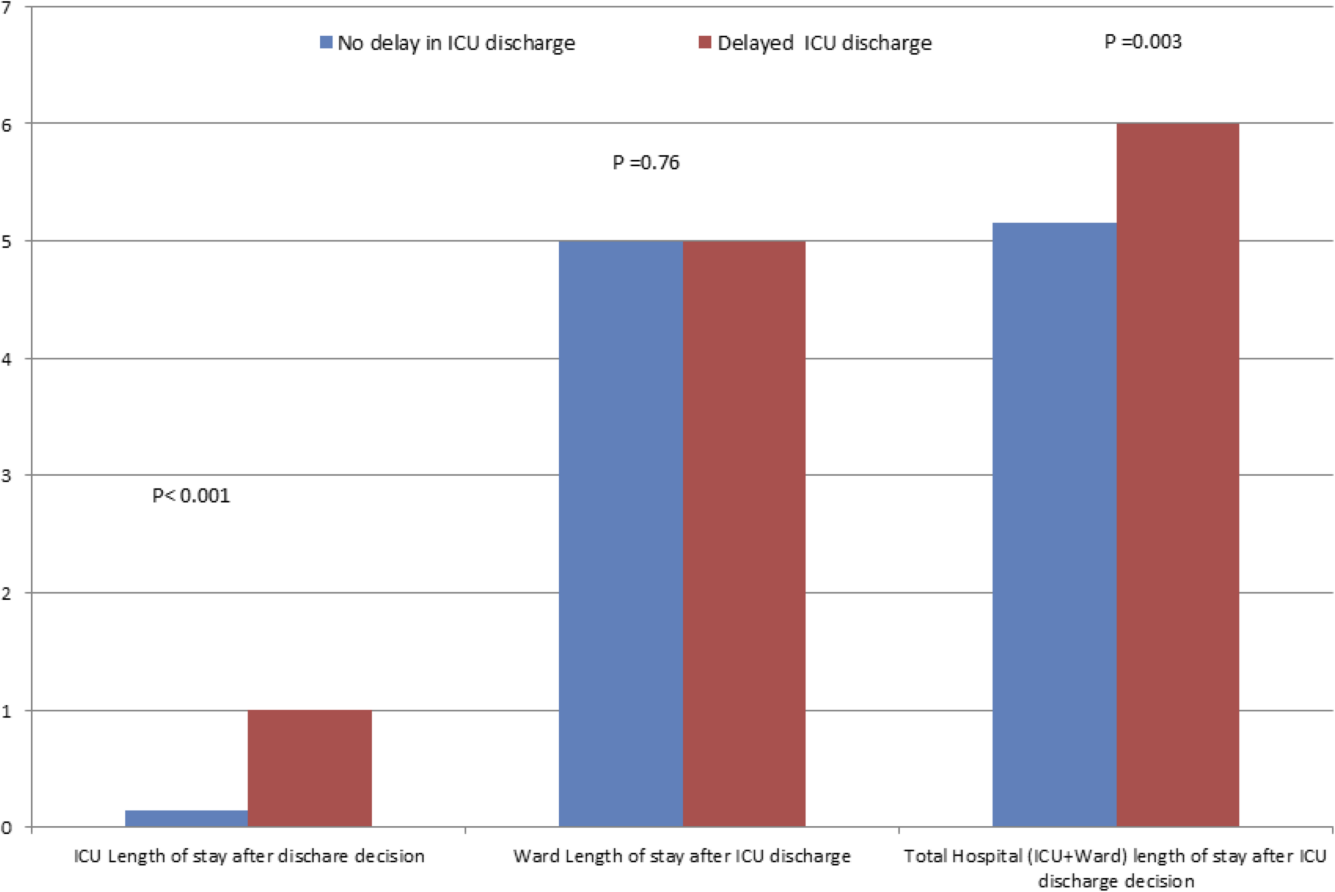
Comparison of ICU, ward and total hospital length of stay between patients with and without a delay in ICU discharge.

In 477 patients (49.9% (95% CI 46.7% to 53.2%)) experienced a delay in ICU discharge. The median duration of the delay was 24 hours (IQR 8.8–31) (Table 2). The most common reason for delay in discharge was the non-availability of ward bed. A small but significant proportion of patients discharge was delayed to avoid overnight discharge. The characteristics at the time of discharge from ICU are comparable in both groups except for the increased need for isolation room in patients with a delay in discharge (Table 3). On univariable comparisons, patients whose discharge was delayed had a significant increase in duration of hospital stay from the time discharge decision was made (Table 4). However, after the patients were discharged to the ward, the hospital length of stay was comparable between both groups (Median days and IQR: 5 (2–9) Vs 5 (2–9); p = 0.76) (Fig 2).

**Table 2:** Comparisons of duration of delay, reasons for delay and discharge decision revoked.

Variable	Discharged with no delay (N = 478)	Discharged with delay (N = 477)	P Value
Duration of stay in ICU after discharge decision, Median hours (IQR)	3.5 (2–4.7)	24.0 (8.8–31)	< 0.001
***Reasons of the delay***			
No Ward Bed		73.6% (n = 351)	
To Avoid Discharge Overnight / after hours		1.7%% (n = 8)	
Awaiting transfer to nursing home, private hospital ward and psychiatry ward or acute ward at a different hospital		1.8% (n = 9)	
Operational reasons		3.1%(n = 15)	
Palliation		1.3%(n = 6)	
Awaiting review by other teams before discharge home		0.4%(n = 2)	
Delay in preparation of ICU discharge summary		0.2%(n = 1)	
Unclear/missing reason		17.8% (n = 85)	

**Table 3:** Characteristics at ICU discharge.

Variable	Discharged with no delay(N = 478)	Discharged with delay (N = 477)	P Value
**Tracheostomy**	0% (n = 0/475)	1% (n = 5/473)	0.26
**Isolation room**	2% (n = 9/472)	5% (n = 23/469)	0.026
**Cardiac Monitoring**	6% (n = 28/474)	7% (n = 33/471)	0.68
**Non Invasive Ventilation**	2% (n = 9/454)	1% (n = 5/460)	0.61
**Total Parenteral Nutrition**	3% (n = 14/451)	2% (n = 9/460)	0.54
**Renal Replacement Therapy**	1% (n = 5/455)	1% (n = 5/459)	0.58
**Glasgow Coma Scale (mean, SD)**	14.81 (0.73) (n = 358)	14.83 (0.66) (n = 377)	0.62
**Treatment Limitations**	11% (n = 49/448)	12% (n = 54/451)	0.71
** Not for CPR**	100% (n = 49)	88.9% (n = 48)	
** Not for Intubation**	93.9% (n = 46)	81.5% (n = 44)	
** Not for RRT**	36.7% (n = 18)	31.48% (n = 17)	
** Not for MET call**	4.1% (n = 2)	5.56% (n = 3)	
** Modified MET call criteria**	12.2% (n = 6)	7.4% (n = 4)	
** Not for Code Blue**	16.3% (n = 8)	12.9% (n = 7)	

**Table 4:** Comparison of primary and secondary outcomes.

Variable	No delay (N = 478)	Delayed (N = 477)	P value
**Primary outcome**			
Duration of hospital stay from the time of ICU discharge decision, days	5 (2–9) (n = 453)	6 (3–10) (n = 455)	0.003
**Secondary outcomes**			
Delirium in ICU day 0	6% (n = 24/397)	7% (n = 28/396)	0.39
Delirium day 1 after ICU discharge decision	-	8% (n = 18/219)	
Delirium day 2 after ICU discharge decision	-	11% (n = 6/54)	
Delirium day 3 after ICU discharge decision	-	10% (n = 2/19)	
Delirium Prevalence in ICU after ICU discharge decision	6% (n = 24/397)	9% (n = 36/397)	0.11
After Hours Discharge from ICU	10% (n = 48/478)	34% (n = 162/476)	< 0.001
Parent-team review in ICU	91% (n = 392)	93% (n = 423)	0.25
Number of reviews by parent-team in ICU	1 (1–1) (n = 431)	1 (1–2) (n = 455)	< 0.001
New Sepsis in ICU	1% (n = 4/438)	1% (n = 5/457)	0.61
Discharge decision revoked	0% (n = 0/443)	1% (n = 5/460	0.51
Unplanned readmissions within 72 hrs	4% (n = 17/418)	2%(n = 9/436)	0.14
Discharged alive % (n)	96% (n = 339/353)	94% (n = 375/399)	0.12
Home% (n)	67% (237/353)	65% (n = 255/393)	
Other hospital% (n)	14% (49/353)	13% (51/393)	
Rehabilitation% (n)	14%(49/353)	14% (55/393)	
Deceased% (n)	3% (11/353)	4% (16/393)	0.3
Delirium (Ward Day 1)	3% (n = 11/364)	4% (n = 14/356)	0.54
Parent–team Review (Ward Day 1)	93% (n = 363/390)	96% (n = 372/413)	0.10
Delirium (Ward Day 2)	3% (n = 11/327)	6% (n = 18/315)	0.15
Parent-team Review (Ward day 2)	93% (n = 333/358)	94% (n = 321/341)	0.65
Delirium Prevalence on Ward	4% (n = 15/369)	6% (n = 19/370)	0.51
Number of Parent Team Reviews on Ward	2 (2–2) (n = 392)	2 (2–2) (n = 393)	0.40
New Sepsis on Ward	2% (n = 8/395)	1% (n = 4/395)	0.34

**Fig 3:**
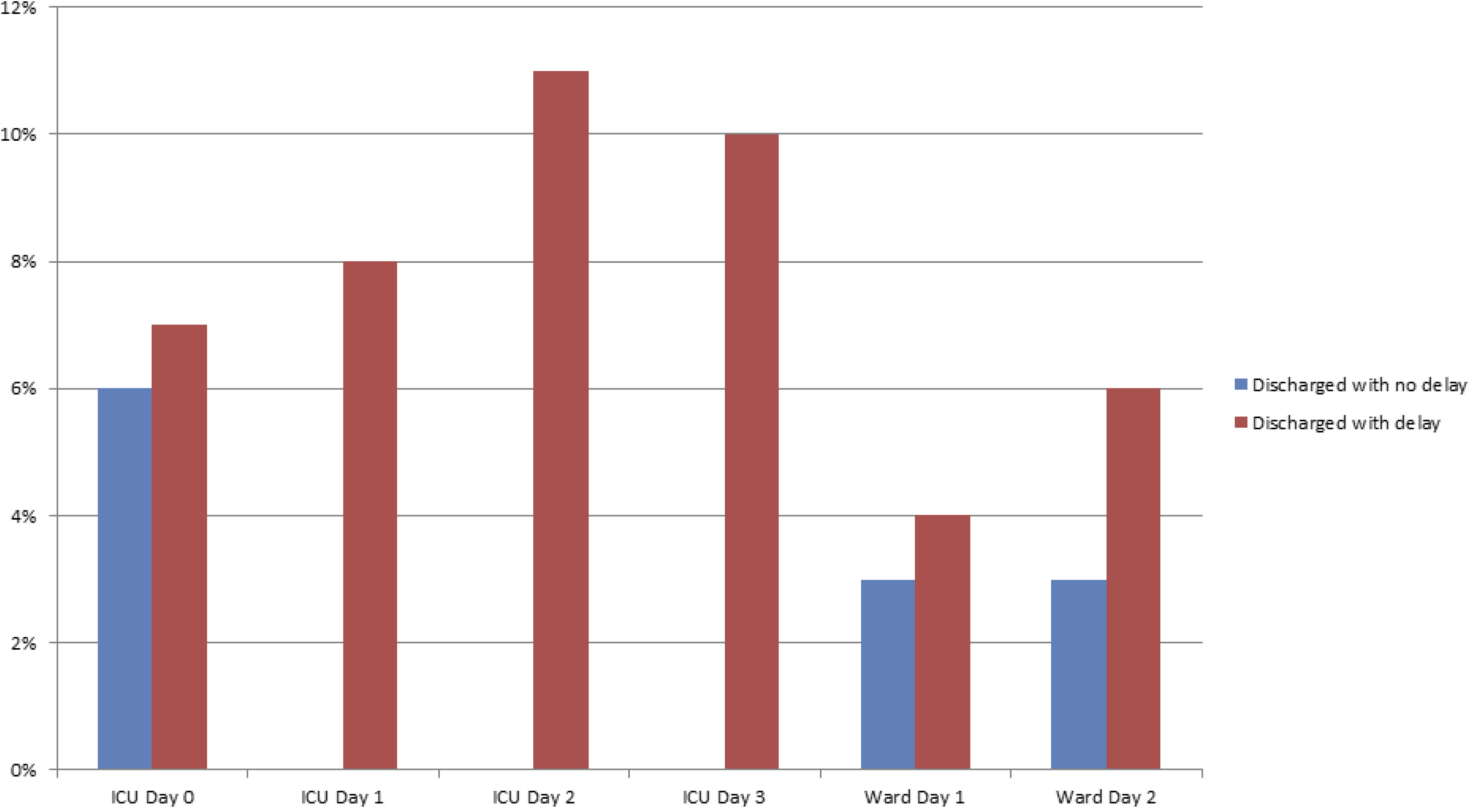
Incidence of delirium after ICU discharge decision to 48 hours in ward.

Comparisons of secondary outcome measures are shown in Table 4. The incidence of sepsis and delirium were comparable in both groups. However, in comparing the subgroup of patients where ICU discharge was delayed by more than 24 hours the incidence of delirium (assessed on the 2^nd^ day of ICU discharge delay) was significantly higher (6% V 11%; p = 0.035) (Fig 3) as compared to those patients who did not have a delay in ICU discharge. After-hours discharge was significantly higher in patients where discharge was delayed (Table 4). There was no significant difference in revoking of discharge decisions between both groups (Table 4). The incidence of delirium and sepsis during the first 48 hrs in the wards were comparable between both groups (Table 4). Patients admitted after major gastrointestinal surgeries had a higher incidence of delay in discharge (Table 5).

**Table 5:** Comparisons of incidence in delay in relation to admitting units.

Admitting Unit	Discharged with no delay (N = 478)	Discharged with delay(N = 477)	P value
Neurology	6% (n = 27)	7% (n = 33)	0.41
Cardiology	6% (n = 28)	5% (n = 24)	0.80
Toxicology/general medicine	6% (n = 28)	9% (n = 42)	0.15
Orthopedic surgery	8% (n = 38)	5% (n = 24)	0.07
Colorectal surgery, GI and hepatobiliary	23% (n = 109)	30% (n = 141)	0.02
Endocrinology	4% (n = 19)	2% (n = 9)	0.09
Urology	2% (n = 9)	1% (n = 5)	0.09
Respiratory medicine	12% (n = 57)	14% (n = 66)	0.43
Renal medicine	3% (n = 14)	1% (n = 5)	0.049
Head and neck Surgery	5% (n = 24)	3% (n = 14)	0.27
Thoracic surgery	3% (n = 14)	2% (n = 9)	0.45
Vascular surgery	3% (n = 14)	3% (n = 14)	0.98
Oncology and haematology	1% (n = 5)	0% (n = 0)	0.73
Obstetrics and Gynaecology	2% (n = 9)	1% (n = 5)	0.11
Not specified or more than one unit	9% (n = 43)	10% (n = 47)	0.72

Multivariable analysis demonstrated that the delay in ICU discharge was independently associated with an increased hospital length of stay after the ICU discharge decision. After adjusting for known confounders, patients who had a delay in ICU discharge had a 1.7 day increase in duration of hospital stay from ICU discharge decision ((Geometric mean 1.57 (95%CI 1.20–2.06) Vs 3.30 (95%CI 2.51–4.35); P<0.001) as compared to patients with no discharge delay. Sensitivity analysis including patients who survived hospital discharge showed a similar increase in duration of hospital stay (from the time of ICU discharge decision) with the delay in ICU discharge (Geometric mean 1.95 (95%CI 1.45–2.61) Vs 3.67 (95%CI 2.72–4.95); P<0.001).

## Discussion

Our study showed that patients who were discharged with a delay of more than 6 hours had a longer length of hospital stay and more patients were discharged after hours. Furthermore, a higher incidence of delirium was noted if their discharge was delayed by more than 24 hours. The most common cause for the delay in discharge was the non-availability of hospital ward bed.

Studies evaluating the reasons for delayed discharge reported similar findings [11–13]. The most common reason reported was lack of ward bed. The other major factor that leads to delay in discharge was medical concerns or clinical deteriorations after the ICU discharge decision [11, 13]. In our study the most common reason for the delay was non availability of hospital bed. However, we have not noted patient deterioration warranting revoking of ICU discharge decision noted in other studies.

The results of our study may aid in planning of health care delivery in settings similar to our study centres. A delayed discharge from ICU is associated with increased length of hospital stay which may be associated increase in health care costs esp. of the intensive care costs. Our study showed that after the patients were discharged from ICU the duration of hospital stay is comparable between patients with or without ICU discharge delay. It is unclear from our study as to the exact reason for this. However, it is likely that the physicians and other health care providers may require a certain amount of time to assess the suitability of patients who may be discharged from the hospital irrespective of the time patients spend in ICU after ICU discharge decision.

Our study found that patients with higher APACHE III score on admission experienced an increase in delay. It is difficult to explain this phenomenon as the severity of illness for our patient cohort at the time of discharge decision making should have been comparable. It is conceivable that when patients were ready for discharge to the wards in the setting of inadequate availability of ward beds, patients with lower APACHE III score (and therefore possibly lower ICU length of stay prior to discharge decision) may have been discharged in preference to those patients with higher APACHE III scores. We did not notice any increase in the hospital duration of stay between both groups after they were actually discharged from ICU. There was also no difference in unplanned readmission within 72 hour rates or revoking the decision to discharge from ICU in either group. Furthermore, delayed ICU discharge did not contribute to a shorter hospital stay post ICU discharge. These findings suggest that patients who were discharged to the ward within 6 hours were appropriate for ward care and a timely discharge may potentially have reduced the duration of hospital stay from the time of ICU discharge.

The delay in ICU discharge may also reduce the availability or delay the access of intensive care facilities for those who are in need. We did not evaluate the incidence of delay in admission to ICU or admission refusal to ICU due to lack of ICU bed when patients were referred for ICU admission. It is possible that during our study period some patients admission to the intensive care may have been delayed due to the discharge delays from ICU. Such delay in admission may increase mortality and morbidity [14–16]

The incidence of delirium associated with ICU stay is reported to be between 30 and 80% [17, 18]. The apparent lower incidence of delirium (6–9%) reported in our study must be interpreted in the light that we only have reported the incidence of delirium after the discharge decision was made to 48 hours of stay in the hospital ward.

Our study demonstrated that the incidence of delirium was higher in patients whose ICU discharge was delayed by more than 24 hours. While age, severity of illness are known to be predictors of delirium during the ICU stay [19] it is not clear if these features will predict the occurrence of delirium after discharge of patients from ICU. Our study revealed that patients who had a delay in discharge were older and had higher APACHE III scores. While it may be possible to attribute this to the increased incidence of delirium, there was no significant difference in delirium in patients ‘at the decision time’ of ICU discharge or on the 1^st^ and 2^nd^ days in the wards (Fig 3, Table 4).

After hours discharge was noted to be significantly higher in patients with a delay in ICU discharge. Some studies have noted an increase in mortality in patients discharged form ICU after hours [2, 3, 20] and other have noted no such increase [21]. As afterhours discharge may be a consequence of a delay in ICU discharge, every effort should be made to reduce the delays in ICU discharge thorough appropriate planning.

### Strengths

Our study is a prospective study that was conducted in 5 intensive care units. These results should be generalisable for intensive care units with similar case mix. The duration of the study is long enough and included data collection during the entire weeks to account for any day to day or weekend fluctuations of admission and discharge process. Investigation of concepts such as delay in discharge from ICU is almost impossible with other study designs such as randomised controlled trails. Observational studies such as this study will contribute to understand the implications of such issues.

### Limitations

It is possible that the delay in discharges may have variation during different seasons or time periods. We did not have predefined criteria to assess the suitability of ICU discharge. However the study’s pragmatic design should assist in quantifying the extent of this problem in routine practice of intensive care medicine in Australia and other comparable health care settings. We did not have the data on the duration of ICU stay before ICU discharge decision or the organ supports and other therapies the patient’s may have received or their severity illness scores, or ICU or ward census data at the time of ICU discharge decision. These data may have given further insight into the reasons for delay in ICU discharge. The assessment of delirium may have been more rigorous and frequent in ICU as compared to ward assessments that may have contributed to an increased increase of delirium in ICU. For those patients who developed delirium in ICU post ICU discharge decision, the presence of delirium by itself may have contributed to the delay in discharge.

### Conclusions

Delayed discharge was noted in nearly 50% of patients. Delay in ICU discharge is associated with an increased duration of hospital stay and the incidence of afterhours discharge.
